# Hospital Meetings

**Published:** 1902-01-18

**Authors:** 


### HOSPITAL MEETINGS.

### NATIONAL HOSPITAL FOR THE PARALYSED.

A SPECIAL general meeting of the National Hospital for the Paralysed and Epileptic was held on the 9th instant, pursuant to the transitional rule passed at the meeting on December 12th ult. The Earl of DUDLEY was voted to the chair.

The first business was the election of a President of the hospital, and on the motion of Mr. BISCHOFFSHEIM, seconded by Mr. MELVILLE GREEN, Lord Dudley was so elected, amid applause. His lordship expressed his sense of the honour done him, and said that though he had not had an opportunity of following the work of the institution very closely in the past yet he had for a long time taken a great interest in it and would spare no effort to carry out the duties of the office.

The next item on the agenda paper was the election of auditors, but Mr. MELVILLE GREEN proposed the postponement of this until the annual meeting in March, since the accounts were already in the hands of auditors, and there would therefore be nothing for a newly appointed firm to do for nearly another year. This was agreed to.

Mr. BOWER, the solictor, asked leave to mention that the question of the alteration of the rules as set out in the fifth item on the agenda paper would, in the altered circumstances that had arisen since it was drafted, not now be proceeded with, and no motion on the subject would be made.

The CHAIRMAN then invited the proposal of names for the election of ten ordinary members of the Board of Management, but mentioned that only those of which notice had already been given could be submitted to the meeting.

Mr. BISCHOFFSHEIM proposed, and Dr. GRIFFITHS seconded, the following 10 gentlemen:—Mr. Ernest de la Rue, Mr. Melville Green, Mr. Macmillan, Sir Alexander McKenzie, Sir John Paget, Mr. Danvers Power, Mr. Arnold Royle, Mr. Edgar Speyer, Rev. Dr. Wace, and Mr. James Wigan.

Mr. H. H. NELSON pointed out that the nominations put forward by the committee of selection eliminated entirely those who had had to do with bringing the institution to the eminent position it enjoyed. There would be no leaven of the old management, and they must remember that at the same time they would be deprived of the services of Mr. Burford Rawlings. He thought that savoured of carrying things too far. They had triumphed—and in his opinion rightly so—but they were not there to execute the vengeance of the medical staff on those they had conquered. Whatever the faults of the old management, it was undeniable that the hospital had prospered; even the report on which these proceedings were founded acknowledged that their affairs on the whole had been wisely carried on. He thought it was not good policy to eliminate the whole of the members of the old board, and he had given notice to propose to add four members of that board. He had to act at short notice, and had had no opportunity to confer with these gentlemen, two of whom had since declined to stand. The two remaining were Mr. Frank C. Capel and Mr. John Pearman, and these he now nominated. Mr. Nelson was proceeding to call attention to the slight and recent qualification from the point of view of monetary subscriptions of the nominees of the committee of selection, when the chairman expressed the hope that he would confine himself to recommending his own candidates without disparaging others.

Mr. CAMPBELL seconded the nomination.

Mr. NELSON called the chairman's attention to a letter handed up to him and asked that it might be read, but the chairman declined to do so, with the remark that it would be very inconvenient to be bombarded with letters from absent governors who, if they were interested, could be present to speak for themselves.

Mr. NETTLEFOLD supported Mr. Nelson's nominees, and suggested that two of the ten new men should retire in their favour. Otherwise he feared the hospital might lose the support of those who might not like so off-hand a way of dealing with the old board.

The CHAIRMAN observed that it was, of course, desirable that the number of pegs should be reduced to the number of holes, but he understood that an attempt had already been made outside the room to arrive at a settlement but had failed.

Before the vote was taken a long discussion arose as to whether the staff as ex officio governors were entitled to vote, the difficulty arising owing to the fact that under the old rules they could not, while under the new rules they could, and it being doubtful whether the new rules were yet in force. Mr. BOWER, the solicitor, expressed the opinion that by the wording of the transitional rule the new rules would not come in force till the next meeting; Mr. MELVILLE GREEN and others were quite clear that, except as regarded the provisional retention of Mr. Burford Rawlings' services while the changes were being effected, the new rules were already in force.

The CHAIRMAN said the point was an important one, and he proposed to take the sense of the meeting on it. But it seemed to him to be not so much a matter of the strict interpretation of the letter of the transitional rule as of a common-sense view of the situation. It seemed to him absurd to hold a meeting to make arrangements under an entirely new constitution, and to govern its proceedings by the rules of the old constitution. He would remind them that the transitional rule as originally drawn enacted that the new rules should come into force at the first general meeting after their adoption, and this was that meeting. But just before their adoption it was realised that since those rules provided for Mr. Burford Rawlings' retirement they would then have no secretary, and that considerable difficulty would thereby be created. Mr. Rawlings was good enough to consent to act during the hiatus between the in-coming and the out-going board of management, showing thereby that despite all that had happened he had a mind big enough to allow the good of the hospital to override any possible rancour that might exist. Words were therefore introduced to provide for this, and it was this alteration which Mr. Bower held prevented the new rules from coming into force. But clearly it was the intention of the framers that the words introduced should apply to the difficulty as to the secretary alone—in all other points they meant the new rules to apply. The position was really this. For some time there had been a most distressing controversy with regard to giving the medical staff a voice in the management. It had at length been decisively decided that they should have such a voice, and it seemed to him absolutely nonsensical to deny them the right to vote in the election of the board of management and so prevent them from having a voice until 1904. He thought it only reasonable that they should adopt the new state of things the committee had laid down, should decline to raise again the old issue, and should let the beginning of that year be the beginning of a new, and, he hoped, a happier era.

The meeting then decided in favour of allowing the staff to vote, and the Chairman thereupon ruled that that course was the proper one to pursue.

Voting for members of the board of management was then proceeded with. Mr. Bischoffsheim's nominees received from 28 to 34 votes each, including 8 ex-officio votes. Mr. Pearman received 16 and Mr. Capel 17 votes, and the Chairman therefore declared the first-named 10 elected.

Mr. Nelson demanded a poll, being supported by the requisite number of governors, and it was arranged that the ballot should be taken at 12 o'clock and the result declared as soon after 2.30 as possible on Thursday, 23rd instant, the Chairman making it clear that the ballot would be governed by the new rules.

A vote of thanks to the Chairman terminated the proceedings.

